# Gears-In-Motion: The Interplay of WW and PPIase Domains in Pin1

**DOI:** 10.3389/fonc.2018.00469

**Published:** 2018-10-25

**Authors:** Yew Mun Lee, Yih-Cherng Liou

**Affiliations:** Department of Biological Sciences, Faculty of Science, National University of Singapore, Singapore, Singapore

**Keywords:** Pin1, WW domain, peptidyl-prolyl *cis/trans* isomerase (PPIase), phosphorylation, interdomain communication, cancer target, drug development

## Abstract

Pin1 belongs to the family of the peptidyl-prolyl *cis*-*trans* isomerase (PPIase), which is a class of enzymes that catalyze the *cis*/*trans* isomerization of the Proline residue. Pin1 is unique and only catalyzes the phosphorylated Serine/Threonine-Proline (S/T-P) motifs of a subset of proteins. Since the discovery of Pin1 as a key protein in cell cycle regulation, it has been implicated in numerous diseases, ranging from cancer to neurodegenerative diseases. The main features of Pin1 lies in its two main domains: the WW (two conserved tryptophan) domain and the PPIase domain. Despite extensive studies trying to understand the mechanisms of Pin1 functions, how these two domains contribute to the biological roles of Pin1 in cellular signaling requires more investigations. The WW domain of Pin1 is known to have a higher affinity to its substrate than that of the PPIase domain. Yet, the WW domain seems to prefer the *trans* configuration of phosphorylated S/T-P motif, while the PPIase catalyzes the *cis* to *trans* isomerasion. Such contradicting information has generated much confusion as to the actual mechanism of Pin1 function. In addition, dynamic allostery has been suggested to be important for Pin1 function. Henceforth, in this review, we will be looking at the progress made in understanding the function of Pin1, and how these understandings can aid us in overcoming the diseases implicated by Pin1 such as cancer during drug development.

## Introduction

Posttranslational modifications (PTMs) introduce diversity to the functions of many proteins in the cellular proteome. This allows the cells to exert more biological functions with lesser number of proteins. There exist many types of PTMs, of which, the reversible phosphorylation is widely studied for its role in regulating many signaling cascade (1). Initially, phosphorylation of a protein was thought to be the final step in activating or inhibiting signaling cascades until the discovery of the group IV WW domain proteins, notably the Pin1 protein (2, 3). Pin1 possesses two major domains, namely the WW domain and the peptidyl-prolyl *cis/trans* isomerase (PPIase) domain (3). The WW domain consists of two highly conserved tryptophan amino acids separated by ~20–22 residues, allowing Pin1 to bind to the phosphorylated consensus site ser/thr-pro (pS/T-P) motif (3–5). This allows Pin1 to exert its molecular function as an isomerase *via* the PPIase domain, leading to the *cis*/*trans* conversion of its substrate to elicit the intended biological outcomes (2, 6). This additional modification on the phosphorylation sites of multiple Pin1 substrates has provided an alternate view on how signaling cascades could be regulated under different cellular conditions (7, 8).

**Figure 1:**
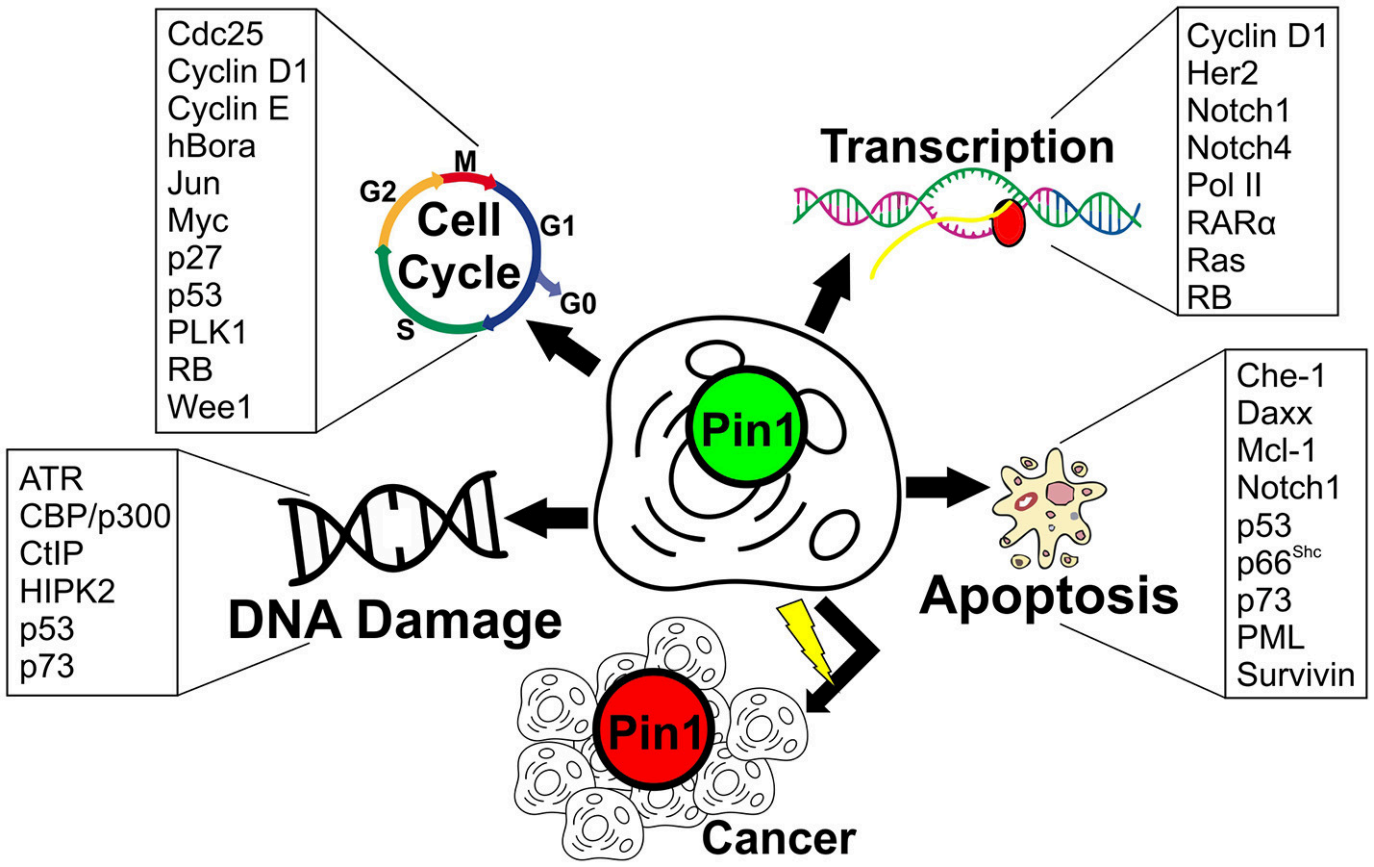
Pin1 substrates belonging to various cellular processes linked to cancer. Cell cycle – Cdc25 (18): Cell division cycle 25; Cyclin D1 (19–21); Cyclin E (22); hBora (23): Human protein aurora borealis; Jun (19, 24): Transcription factor AP-1; Myc (25): Myc proto-oncogene protein; p27 (26); p53 (27, 28); PLK1 (6, 29): Polo-like kinase 1; RB (30): Retinoblastoma-associated protein; Wee1 (31). Transcription – Cyclin D1 (19); Her2 (32, 33): Human epidermal growth factor receptor 2; Notch1 (34, 35): Neurogenic locus notch homolog protein 1; Notch4 (35): Neurogenic locus notch homolog protein 4; Pol II (36): RNA polymerase II; RARα (37): Retinoic acid receptor alpha; Ras (38); RB (30). DNA damage-ATR (39): Ataxia telangiectasia and Rad3-related protein; CBP/p300 (24): CREB-binding protein/p300; CtIP (40): C-terminal-binding protein (CtBP)-interacting protein; HIPK2 (41): Homeodomain-interacting protein kinase 2; p53 (27, 28); p73 (42). Apoptosis-Che-1 (43): Apoptosis-antagonizing transcription factor (AATF); Daxx (44): Death domain-associated protein 6; Mcl-1 (45): Induced myeloid leukemia cell differentiation protein; Notch1 (34, 35); p53 (27, 28); p66^Shc;^ (46):66 kDa proto-oncogene Src homologous-collagen homolog (Shc) adaptor protein; p73 (42); PML (47): Promyelocytic leukemia protein; Survivin (47).

So far, Pin1 has been extensively studied for its role in various cellular functions, particularly in cell cycle regulation (2, 6, 9–11). Besides its importance in cell cycle progression, Pin1 has been further implicated in many biological processes such as embryonic development, cell motility, immune responses, gene transcription, and apoptosis (12–17). Due to its diverse role, perturbation to Pin1 expression levels has been implicated in many diseases such as cancer, and neurodegenerative diseases like Parkinson's and Alzheimer's disease (10, 15). Especially in the case of cancer, many functional substrates of Pin1 have been identified to potentially contribute to the manifestation of cancer via various biological processes as summarized in Figure 1. As such, Pin1 has been identified as an important target for therapeutic intervention for these diseases (48–50). Indeed, many Pin1 inhibitors have been identified, with the most recent ones being API-1, KPT-6566, compound 1 and 8 (51–54). Unfortunately, there remains much to do before any breakthrough in targeting Pin1 for the treatment of various diseases is achieved. This stems from our limited understanding of Pin1 mechanism in these biological processes, and how the unique WW and PPIase domains of Pin1 work to elicit its function.

In addition, there have been many structural and protein dynamic studies to understand how Pin1 could target its biological substrates to exert its intrinsic *cis*/*trans* isomeric activity. This would then trigger its intended downstream cellular signals and effects. Many models have been introduced to explain the molecular mechanism of how Pin1 exert its catalytic activity. However, there has not been a model in agreement to truly explain how Pin1 acts on its biological substrates. Therefore, in this review, we will highlight the progress of Pin1 research in elucidating the actual mechanism of Pin1, with an emphasis on the structural importance of Pin1 on its function, and how the perturbation to this fundamental structure could explain for its roles in diseases such as cancer. We will also discuss how these structural features could be used in the drug development of Pin1 inhibitors.

## Detailed structural features of human pin1

**Figure 2:**
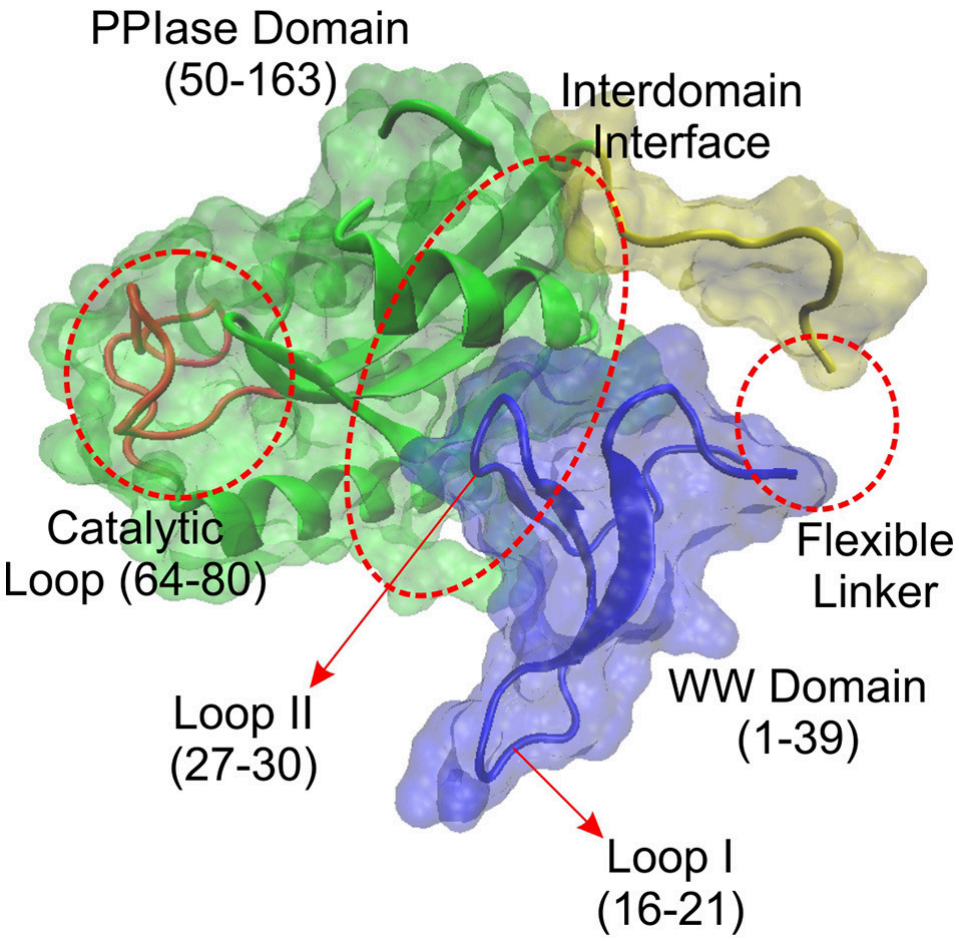
Molecular structure of human Pin1 (PDB: 1PIN) without ligand binding. Pin1 is predominantly made up of two major domains: WW domain and PPIase domain. Within the WW domain, there exist two loops, namely Loop I and II. As for the PPIase domain, there is a catalytic loop at the catalytic site. There also exists PPIase binding domain where it could bind to the pS/T-P motif apart from the WW domain. In addition, existing between the two domains, there lies an interdomain interface that has been found to be important in Pin1 allosteric regulation.

The human Pin1 consists of a total of 163 amino acid residues that forms two distinct domains, the WW domain and the PPIase domain as mentioned previously (Figure 2). The WW domain spans the first 39 amino acid residues of the Pin1 protein, while the PPIase domain spans residues 50–163. Both domains are known to be able to bind to the pS/T-P motifs, with the WW domain binding being noncatalytic in nature, while the PPIase domain possesses the sole catalytic site of the *cis*/*trans* isomerase activity in Pin1 (55–57).

Apart from the two major domains, the Pin1 protein also consists of a flexible linker region consisting of approximately 17 amino acid residues spanning from residue E35 to A53 (58). In addition, within the WW domain, there exist two loops. Loop I is situated at residue S16 to R21, and Loop II at residue H27 to N30 (58). As for the PPIase domain, it also displays two main components. They are the substrate recognition segment (residue K63 to R80) where residues K63, R68, and R69 create a positive charged phosphate-binding loop to facilitate binding to the pS/T-P motif (58, 59). The other important segment of the PPIase domain is the catalytic active site that consists of amino acid residues such as H59, S115, C113, L122, M130, F134, T152, and H157 (58, 60, 61).

Besides these characteristics of Pin1, there exist the presence of a domain interface within Pin1, consisting of amino acid residues I28, the WW domain Loop II (H27 to N30), and part of the PPIase domain (S138 to R142). This domain interface has been suggested to play an important role in interdomain communication that regulates the function of Pin1 upon substrate binding (60–62). These minor features of Pin1 suggest the complexity of Pin1 function upon substrate binding and are a potential reason why Pin1 can interact with a large and diverse number of biological substrates to regulate cellular functions. However, how these characteristics can work hand-in-hand to confer the functions of Pin1 on its substrates remain a largely unfinished work.

## The proposed models of the WW domain and PPIase domain in pin1 substrate interactions

**Figure 3:**
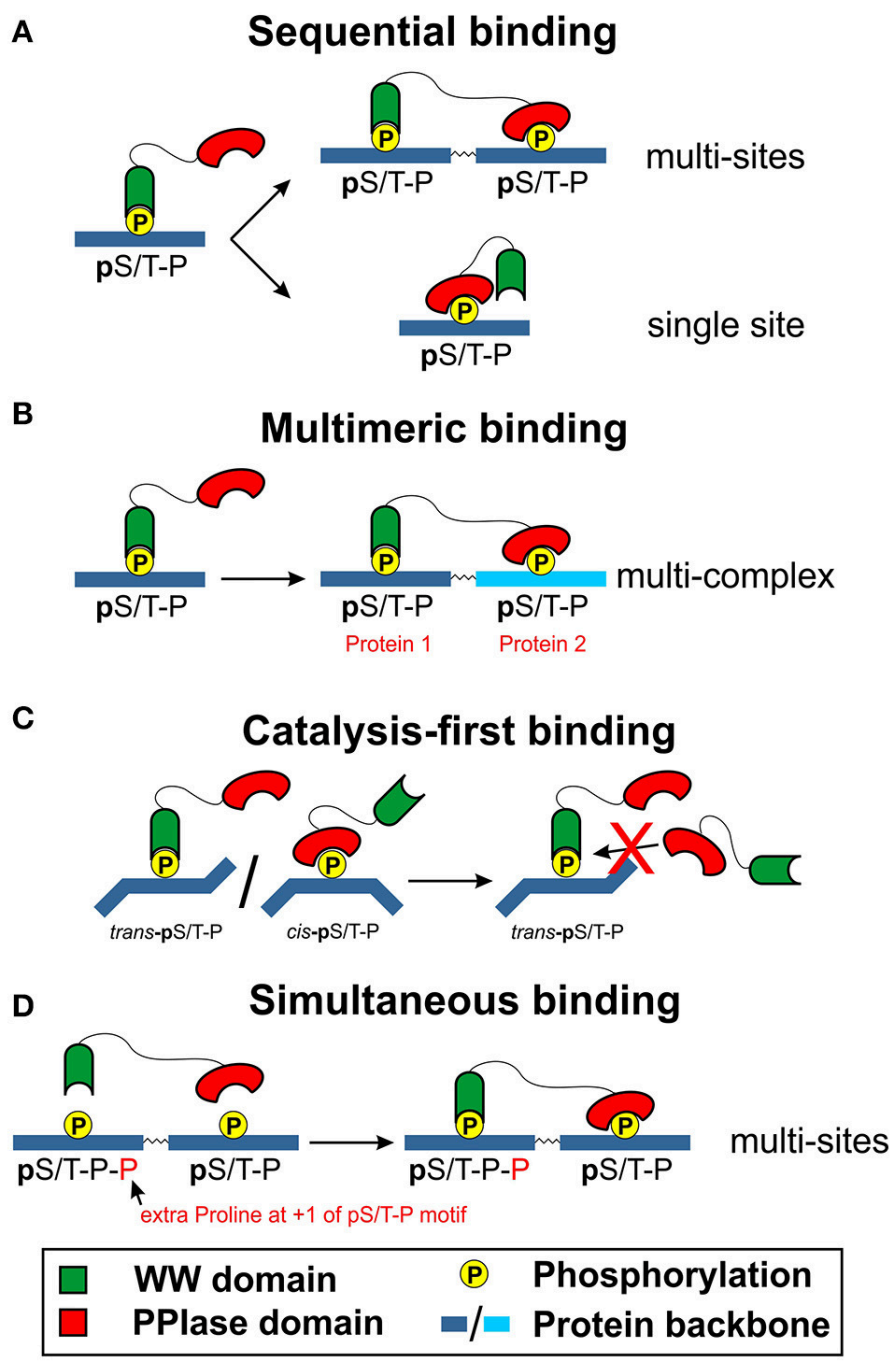
Proposed models of WW domain and PPIase domain interaction with the substrate of Pin1 [first highlighted by Innes et al. (65)]. Two decades of research has shed light on the potential mechanism as to how the WW and PPIase domains could be involved in substrate binding of Pin1. **(A)** Sequential binding model. The sequential binding model involves the initial binding of the WW domain of Pin1 before the PPIase domain could bind to the same pS/T-P motif or another pS/T-P motif on the same protein. **(B)** Multimeric binding model. The WW domain first binds to an active kinase with a pS/T-P motif before being brought close to a substrate of Pin1. The active kinase then phosphorylates S/T-P motif to allow the PPIase domain to bind to it to initiate the isomeric reaction. **(C)** Catalysis-first binding model. Existing *trans* pS/T-P motif will be bound to the WW domain of Pin1, while the PPIase domain could bind to the *cis* pS/T-P motif to catalyze the isomeric reaction to the *trans* configuration. Subsequently, the WW domain would then bind to the new *trans* pS/T-P motif to prevent the reverse conversion. **(D)** Simultaneous binding model. Both WW domain and PPIase domain can bind simultaneously, with the WW domain binding to pS/T-P-P motif and PPIase domain binding to the pS/T-P motif.

As the two main domains of Pin1 protein, much research has been carried out on both WW and PPIase domains to understand their role in the interaction of Pin1 with its biological substrates. It has been reported in the early stages of Pin1 research that the WW domain has a ten-fold higher binding affinity with phosphorylated peptides *in vitro* as compared to the PPIase domain (3, 63). This property of the WW domain in Pin1 has led to a proposition that its major role is to aid Pin1 in specific targeting of its substrate, as well as to increase local concentration of Pin1 substrate to exert its catalytic function (7, 64). This scenario gave rise to a few proposed models of Pin1 substrate interactions. The first proposed model is the sequential binding model of Pin1 (Figure 3A) and remains widely accepted. In this model, the WW domain would first bind to the pS/T-P motif on its target substrate. This allows the PPIase domain to bind with another pS/T-P motif that is present on the same target substrate. Alternatively, the binding of the WW domain would lead to structural conformation change that allows the PPIase domain to displace the WW domain to bind to the same pS/T-P motif to exert its isomeric activity.

In the multimeric binding model (Figure 3B), it is applied in multi-protein complexes containing a Pin1 biological substrate and an active kinase that is present to phosphorylate the S/T-P motif of the Pin1 substrate (66). In this configuration, Pin1 first binds to the kinase at the pS/T-P motif *via* its WW domain. Subsequently, the active kinase would phosphorylate the S/T-P motif on the target substrate of Pin1. As Pin1 is already near its substrate, the PPIase domain would recognize and bind to the pS/T-P motif on the target substrate to exert its isomeric activity. This model is further supported by the identification of Pin1 interactors which are known kinases themselves as reflected in the IntAct Molecular Interaction database (67).

In another model termed the catalysis-first binding model (Figure 3C), it is believed that the phosphorylation of S/T-P motif on the target substrate for WW domain binding requires the PPIase function of Pin1 (57). This proposition was put forth by the observation that in all the known structures of the WW domain bound substrate peptides, the identified binding site is present in a *trans* configuration state (56, 57, 68). Moreover, a study conducted by Verdecia et al. (56) seems to suggest the strict preference of the wild type WW domain of Pin1 to bind to the *trans* configuration of its substrate peptide. Of interest, Pin1 also enhances the dephosphorylation activity of protein phosphatase such as PP2A, which too requires the pS/T-P motif to be in the *trans* configuration state. Therefore, the binding of the WW domain of Pin1 might be a stabilizing action for the Pin1 substrate to remain in its *trans* configuration state for dephosphorylation to occur (69, 70). In addition, a study by Namanja et al. (60) found the preference of WW domain binding to the *trans* but not the *cis* configuration of pS/T-P motif. Thus, if the WW domain prefers binding to a *trans* configured substrate peptide, the PPIase domain of Pin1 would first bind to the *cis* configured pS/T-P motif to catalyze the *cis*/*trans* isomerization to the *trans* configuration. This would lead to WW domain binding to the now *trans* configured pS/T-P motif to prevent the reverse isomerization to occur. This process would then allow the local concentration of the Pin1 PPIase to initiate further *cis*/*trans* isomerization to fast forward the propagation of downstream cellular signaling of the stabilized Pin1 substrate.

Indeed, previous studies have shown that the PPIase domain of Pin1 is able to bind to pS/T-P motif instead of just its WW domain (56, 58, 65). As mentioned earlier, this binding occurs at the substrate recognition segment, in particular, the phosphate-binding loop created by the three amino acid residues of K63, R68, and R69. Furthermore, a study done by Innes et al. (65) demonstrated the importance of this phosphate-binding loop in the target binding of Pin1 to initiate its catalytic isomerase function. This is despite the reported higher binding affinity of the WW domain of Pin1 and reinforced by the presence of PPIase domain-specific Pin1 inhibitors that do not interact with the WW domain (71). As such, the authors suggest that Pin1 could interact with its biological substrate *via* the simultaneous binding model (Figure 3D). In their study, they noticed that pS/T-P motifs that have an addition P residue in the +1 position, pS/T-P-P, seem to be targeted by the WW domain but not the PPIase domain of Pin1. This led them to believe that a substrate with multiple phosphate binding sites could allow for the simultaneous binding of Pin1 to its substrate. They observed this in the binding of Pin1 to Cdc25 and Serine/Threonine-protein kinase (PLK1). On the other hand, they identified that Pin1 binding *via* the PPIase domain is sufficient in proteins with only a single pS/T-P motif such as non-POU domain-containing octamer-binding (NONO) protein and splicing factor, proline-, and glutamine-rich (SFPQ) protein for its activity. As such, there seems to be two types of Pin1 interactors that requires either the binding of both WW and PPIase domains, or just the WW/PPIase domain alone.

These proposed four models of Pin1 interaction with its biological substrates for functional regulation might suggest two aspects. Firstly, there is still lack of concrete evidences to highlight which model(s) Pin1 may deploy for its interaction with its biological substrates. Secondly, these models demonstrate the potentially diverse interaction that Pin1 has on its already diverse biological interactors to play a role in the regulation of various biological functions. All these models do have their merits as well as potential doubts as to its suitability. For instant, the sequential binding model does fulfill the characteristics of the WW domain having a higher affinity of peptide binding to the pS/T-P motif than the PPIase domain. In proteins with multiple sites of pS/T-P motifs, this model could explain the use of the WW domain to localize the concentration of the PPIase domain for isomerization. However, for substrate with a single pS/T-P motif, the potential release of the WW domain from this motif, followed by the binding of the PPIase domain for catalytic activity does not seem to be energy favored. This is also highlighted by studies that we have mention previously (56, 57, 60, 68–70), where the WW domain favors the *trans* over *cis* configuration. As such, the PPIase isomerization would not have occurred with this sequential binding.

For multimeric binding to an active kinase *via* the WW domain of Pin1, the increased local concentration of Pin1 would allow the binding to its biological substrate in the vicinity to elicit its downstream biological processes. Furthermore, the pS/T-P motif of the kinase could be in a *trans* configuration state to allow for WW domain binding. In addition, the catalysis-first binding model could also be associated with the multimeric binding model where Pin1, bound to the active kinase *via* the WW domain, is brought to its target substrate by the active kinase. Subsequently, the active kinase would phosphorylate the Pin1 substrate, allowing for the PPIase domain of the Pin1 to bind and catalyze the *cis-trans* conformational change. Furthermore, the suggested simultaneous binding model could also fit the multimeric binding model, although this would occur on a single protein, instead of the proposed protein complex in the multimeric binding. Of interest, the binding of the WW domain in the *trans* configured pS/T-P motif could lead to conformational changes to the PPIase domain, increasing its binding capacity to the *cis* configured p-S/T-P motif and subsequently its catalytic efficiency. The potential role of interdomain communications between the WW domain and the PPIase domain of Pin1 has indeed been studied in recent years as discussed in the next section.

## Interdomain communications between WW domain and PPIase domain of pin1

As highlighted earlier, Pin1 is made up of two distinct major domains in WW and PPIase domains. Besides these two domains, there exist an internal conduit of hydrophobic residues cluster that connects the interdomain interface and the catalytic site of the PPIase domain (60, 62). This has led to the suggestion of an allosteric mechanism present in Pin1. Classic allostery stems from the binding of a ligand to a part of a protein where it is distal to the active site. This binding would then lead to global conformation change of the protein, or localized changes to its active site, resulting in increased binding affinity of the active site or its enzymatic activities (72, 73). Recently, the concept of allostery, termed dynamic allostery, has been looked into to explain for the absence of structural conformation changes under allosteric effect of ligand binding on the main protein (74–76) since its first introduction more than three decades ago (77). This absence of structural change is replaced by the use of the protein's interdomain communications as demonstrated by computational studies (78, 79) and reviewed elsewhere (80). Indeed, a study by Behrsin et al. (59) showed that the WW domain is essential for the function of Pin1 isomeric activity *in vivo*, suggesting the presence of interdomain communication. In addition, studies conducted on Pin1 and tau interactions highlighted the need of WW domain interaction with the pS/T-P motif for the effective function of the PPIase activity (63, 81). Therefore, understanding the interdomain communication in Pin1, and how this could lead to the dynamic allostery of Pin1 has been of much focus in recent times.

Much work in understanding the presence of interdomain communication and dynamic allostery of Pin1 has been well studied using nuclear magnetic resonance (NMR) studies (82). As highlighted in a review by Sudol and Hunter (83), and studied by Verdecia et al. (56), both Cdc25c and FFpSPR [artificial substrate derived from peptide libraries screening for optimal PPIase efficiency (84)] have higher affinity to the WW domain of Pin1. This is also reflected in other Pin1 substrates that have been identified (66). This higher affinity of the WW domain for Pin1 substrate is contributed by the Loop I structure of this domain, where modification of the Loop I sequence led to the reduction of substrate binding affinity to Pin1 as demonstrated by Peng et al. (85) using the pCDC25C and FFpSPR peptides. Yet, by using a *trans*-locked inhibitor and a *cis*-locked inhibitor synthesized by Wang et al. (86, 87), Namanja et al. (60) found that the isolated PPIase domain has 2–4 times higher affinity to the *cis*-locked inhibitor than the full-length Pin1, while the *trans*-locked inhibitor can be bound by both domains as mentioned previously. This higher binding affinity of the PPIase domain without the WW domain suggests that the removal of the WW domain seems to remove the restriction imposed by the unbound WW domain on the PPIase domain for the *cis* configured pS/T-P motif. This observation clearly suggests a potential form of allostery present in Pin1.

Indeed, data obtained from studies by Namanja et al. (60, 62) showed that the changes in side chain dynamics instead of large structural changes are a means of allosteric communication within the PPIase domain (82). This was further supported using molecular dynamics (MD) simulations (79, 88–90). These changes in side chain dynamics could dictate the flexibility of the PPIase domain to allow for greater substrate binding or enzymatic activity. Base on this side chain dynamics study of Pin1, Peng and co-workers identified the I28 residue as an important amino acid within a conserved hydrophobic conduit of amino acid residues that is affected by substrate binding of Pin1 (62). The subsequent study based on the mutation of I28 to I28A by Wilson et al. (61) led to the discovery of interdomain communications governed by I28, the WW domain Loop II, and the PPIase domain residues S138 to R142. The authors highlighted the influence of this interdomain communications on the side chain dynamics of the conserved hydrophobic conduit region that would affect PPIase domain binding and catalytic activity. This study is further supported by a recent MD study from Barman and Hamelberg (91). They highlighted that the binding of the substrate compacts the WW domain closer to the catalytic domain *via* a hinge-like movement, an observation echoed by a more recent study done by Campitelli et al. (92) using their dynamic flexibility index analysis.

**Figure 4:**
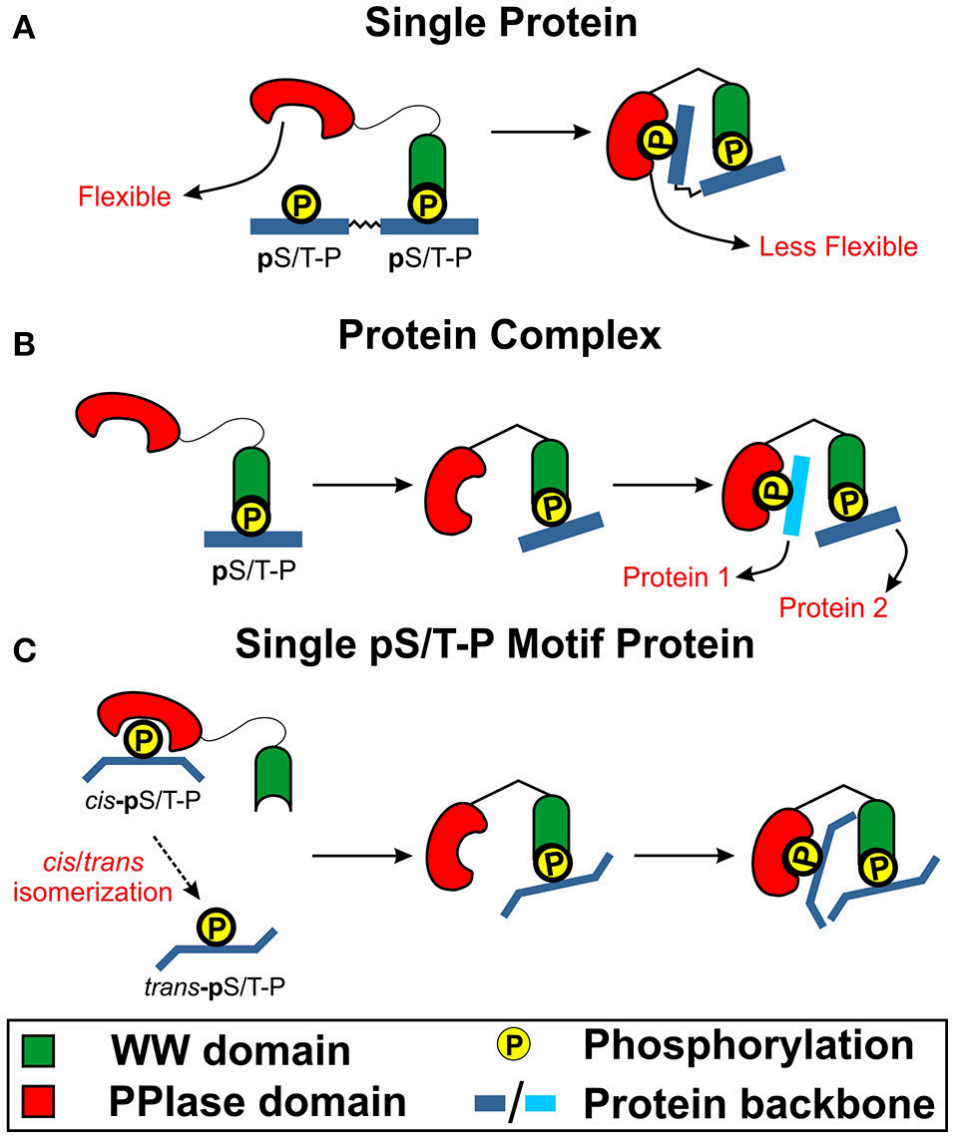
The role of interdomain communication in Pin1 function. Based on recent findings, WW domain could be required for initial binding to initiate conformation changes to the PPIase domain for its binding and catalytic function. **(A)** WW domain binds first to pS/T-P motif that leads to interdomain communication to trigger conformation change (decreased flexibility) that lead to PPIase binding to a distal pS/T-P motif on the same protein to initiate catalytic function. **(B)** WW domain first binds to the pS/T-P motif to initiate interdomain communication to trigger PPIase domain conformation change to bind a pS/T-P motif on another protein to induce catalytic function. **(C)** PPIase domain could first catalyze a few pS/T-P motifs from the *cis* to *trans* configuration. This would lead to WW domain binding and initiate interdomain communication to trigger PPIase conformation change. This would then increase the activity of PPIase domain activity to lead to more cis to trans isomerization of the pS/T-P motif of the same protein in the vicinity.

Collectively, these studies highlight the fact that the binding of WW domain could be crucial in transmitting an intra-protein signal to the PPIase domain and the catalytic site *via* the interdomain contact as highlighted previously. This transmission of signal could be the result of side chain dynamics of the conserved hydrophobic conduit region. This could subsequently lead to subtle hinge-shift mechanism induced by the bound WW domain that leads to improved PPIase domain binding and catalytic activity. Based on these evidences as well as the four binding models of Pin1 to its substrate, the multimeric and simultaneous binding models are the potential model of how Pin1 works in biological signaling cascades. This is due to the potential need of a bound WW domain for Pin1 binding and catalytic activity. Hence, having the WW domain bound to an active kinase or a simultaneous binding of a WW domain followed by the binding of the PPIase domain would support the presence of Pin1 interdomain allostery (Figure 4).

In contrast, there are known examples of Pin1 that only contains one half of the two domains structure. For instant, Pin1At, identified in the plant *Arabidopsis thaliana*, only contains the PPIase domain but not the WW domain as opposed to Pin1 (93). In addition, another single-domain Pin1, known as TbPin1, was identified from the parasite *Trypanosoma brucei* (94). TbPin1 is also known to only contain the PPIase domain. Despite the absence of the WW domain, Pin1At was enough to rescue the temperature-sensitive mutation of Pin1 homolog gene (*ESS1*/*PTF1*) in *Saccharomyces cerevisiae* from death (95). Furthermore, our lab previously showed that TbPin1 alone was also able to rescue the same temperature sensitive *S. cerevisiae* mutant from death (96). These studies highlight that the PPIase domain alone is enough to recover the loss of Pin1 function, brining into question the importance of WW domain in Pin1 function. In hindsight, the WW domain of Pin1 could possess an alternative role in maintaining Pin1 function and could be seen in a complementary role to accelerate the effect of Pin1 function. As discussed earlier in the review, the preference of WW domain for the *trans*-configured substrate of Pin1 potentially suggest that the WW domain of Pin1 seeks to stabilize the *trans* population of the target substrates, leading to increased concentration of the PPIase domain in the vicinity and accelerating the effect of Pin1. Nonetheless, the highlighted studies of TbPin1 and PinAt needs to be replicated in mammals before conclusions could be made to obsolete the role of WW domain in Pin1 function.

## Implications of pin1 as a therapeutic target in cancer

As mentioned previously, Pin1 has been extensively studied due to its diverse involvement in many signaling pathways that has many implications in various diseases, with great emphasis on cancer and neurodegenerative diseases. Despite many studies done on understanding the mechanism of Pin1 interactions with its biological substrates, there remains a gap as to how the understanding of Pin1 mechanism could lead to clinical translation. The most obvious application would be to identify or synthesize inhibitors that could block the functions of Pin1. However, the fact that Pin1 has so many interactors brings the problem of off-target effects. Indeed, the regulation of Pin1 in cancer against that in neurodegenerative diseases is inversely related (97). Therefore, targeting just Pin1 might not be specific enough to reduce the pathogenesis of these diseases.

The development of Pin1 inhibitors has remained a challenge in the field of cancer studies as many known inhibitors of Pin1 remains unspecific enough to only block the effects of Pin1 despite a number of Pin1 inhibitors already found (49, 98, 99). Yet, we cannot rule out the fact that evidences point to the potential key role of Pin1 in tumourigenesis (49, 99, 100). As such, there are continued efforts to identify new and novel Pin1 inhibitors. This is observed from recent efforts to identify various types of Pin1 inhibitors that targets the PPIase catalytic domain (51–53, 101, 102). For instant, a study by Pu et al. (51) identified a chemically synthesized small molecule API-1 that has an Pin1 inhibition concentration (IC_50_) of 72.3 nM, which is 100× less than the IC_50_ of the first Pin1 inhibitor juglone. API-1 was found to bind to the PPIase domain of Pin1. The authors went on to show that API-1 was able to suppress the proliferation of hepatocellular carcinoma cells as well as inhibiting the tumor growth of xenografted mice. These evidences demonstrated the therapeutic value of API-1 as a cancer drug candidate. Another study by Cui et al. (102) demonstrated the use of a novel strategy to develop anticancer agents by inhibiting Pin1 activity *via* the synthesis of various pyrimidine derivatives. The authors were able to identify four potential Pin1 inhibitors with IC_50_ of <3 μM. Furthermore, this type of drug is only the second covalent binding Pin1 inhibitors to the phosphate-binding loop in the PPIase domain besides juglone. These studies clearly highlight the potential therapeutic value of Pin1 as a cancer drug target. Unfortunately, no drugs thus far has reached clinical trials (49, 98).

Of interest, most drugs identified thus far are mainly targeting the PPIase domain, especially the catalytic domain, with few drug candidates found to target the WW domain (98) as summarized in the list of known Pin1 inhibitors discovered thus far in Table 1. For instant, a series of studies conducted by Murray's group (107, 108, 115) set out to identify novel Pin1 inhibitors that are non-phosphorylated small molecule inhibitors that target the Pin1 active site at the PPIase domain using a structure-based design approach. They focused on finding a Pin1 inhibitor that binds to the phosphate-binding loop and were successful in first identifying benzothiophene with sub-micromolar inhibitory activity on Pin1 (107, 108). Unfortunately, this class of inhibitor showed low binding affinity to Pin1 when tested with whole cell assay. This led to the authors to suggest that its low cellular permeability could be the cause of such poor *in vivo* binding affinity to Pin1. The authors went on to improve the permeability of the benzothiophene class of drug to eventually yield dihydrothiazoles as a potent Pin1 inhibitor with low micromolar inhibitory activity toward cancer cell proliferation (115). However, the improvement of permeability led to reduced Pin1 inhibitory potency. This brings a question whether cellular permeability could be major limitation in their development of these structure-based Pin1 inhibitors targeting the PPIase domain.

**Table 1:** Selected known Pin1 inhibitors shown to block Pin1 function by targeting various part of Pin1 protein [adapted from Zhou and Lu (49)].

**Inhibition Site**	**Pin1 inhibitor**	**Mode of discovery**	**IC_50_**	**Mechanism of action**	**System tested**	**Limitation(s)**
Pin1 active site	Juglone (103)	Low-throughput enzymatic (PPIase) assay	-	Covalent modification of Cys in the active site	*in vitro*	Low specificity
	PiB (104)	Low-throughput enzymatic (PPIase) assay	1.5 μM	-	*in vitro*; cell lines	Little evidence of Pin1 binding; insoluble in DMSO
	Pepticinnamin analogs (104)	Combinatorial synthesis	600 nM	-	*in vitro*	Inconclusive evidence of Pin1 binding
	*Cis*-locked alkene peptidomimetics (87)	Structure-based design	1.5 μM	Bind to Pin1 active site via substrate mimicking	*in vitro*; cell lines	-
	D-peptide inhibitor such as Ac-Phe-D-Thr(PO_3_H_2_)-Pip-Nal-Gln-NH_2_ (68, 105)	Solid phase peptide library synthesis	As low as 1 nM	Competitive inhibitor of Pin1 active site	*in vitro*; cell lines	Inactive in cell lines
	Aryl indanyl ketones (106)	Structure-based design	As low as 200 nM	Reversible inhibitor of Pin1 active site undergoing “twisted-amide” transition state	*in vitro*; cell lines	Binding to Pin1 not as well as hypothesized
	Benzothiophene (107, 108)	Structure-based design	6 nM	Binds to Pin1 active site with high specificity	*in vitro*; cell lines	Potential low permeability; inactive in cell lines
	Phenyl imidazoles (109, 110)	Structure-based design	830 nM	Binds to Pin1 active site	*in vitro*; cell lines	Some variant inactive in cell lines
	ATRA (all trans retinoic acid) (111)	High-throughput mechanism-based screening	800 nM	Binds to Pin1 active site via substrate mimicking	*in vitro*; cell lines; mouse models; APL human patients	Moderate efficacy in humans; short half-life in humans
	KPT-6566 (52)	High-throughput structural- and mechanism-based screening	1.2 μM	Covalent binding to Pin1 active site at C113 specifically	*in vitro*; cell lines	-
	Pyrimidine derivatives (102)	In-house library screening	As low as 1.68 μM	Covalent binding to the binding pocket of Pin1 active site	*in vitro*; cell lines	-
PPIase domain	Dipentamethylene thiauram monosulfide (112)	Protease coupled enzymatic (PPIase) assay	50 nM	Competitive inhibitor of Pin1 PPIase domain	*in vitro*; cell lines	Possible low specificity
	Halogenated phenyl-isothiazolone TME-001 (113)	Real-time fluorescence detection method	6.1 μM	Competitive inhibitor of Pin1 PPIase domain	*in vitro*; cell lines	Possible low specificity
	Cyclic peptide inhibitor Cys-Arg-Tyr-Pro- Glu-Val-Glu-Ile-Cys (113)	Phage display screening	500 nM	Competitive inhibitor of Pin1 PPIase domain	*in vitro*	Cannot be used to inhibit intracellular Pin1 activity
	API-1 (51)	Computer-aided high-throughput virtual screening	72.3 nM	Binds to Pin1 PPIase domain specifically	*in vitro*; cell lines; mouse models	-
WW domain	EGCG (epigallo-catechin-3-gallate) (114)	Phenotypic association	20 μM	Bind to both WW and PPIase domains	*in vitro*; cell lines; mouse models	No reports of inactivation on isolated PPIase domain

Of importance, these studies only made use of the PPIase domain as the main consideration in designing of their structure-based Pin1 inhibitor. The authors did highlight some important points supporting intra-structure dynamics of the whole Pin1. Firstly, they noticed that despite the rich hydrogen bonding potential within the phosphate-binding loop in the PPIase domain of Pin1, hydrophobic interactions were central to benzothiophene binding to Pin1. Secondly, they also highlighted the presence of H-bond formation beyond the phosphate-binding loop that could be important for the Pin1 inhibitor binding infinity. Lastly, they highlighted the high degree of flexibility in Pin1 active site interaction with the inhibitors. All these observations do align with our previous discussion where interdomain communications between WW and PPIase domains could affect the side chain dynamics on the conserved hydrophobic conduit region and alters PPIase domain binding affinity (61, 91, 92). Moreover, we previously mentioned that the removal of the WW domain could alleviate the restriction imposed by the unbound WW domain on the PPIase domain for the *cis* configured pS/T-P motif (60). Henceforth, in line with these observations, much remains to be done to utilize the information from the studies of the actual roles by the WW and PPIase domains of Pin1, including the potential impact of interdomain communication could play in future drug screening for Pin1 inhibitors.

As suggested from all the studies on the two domains of Pin1 covered in this review, the WW domain binding displays a potentially important role in driving the signaling cascade triggered by Pin1 catalytic function. To advance our development of promising Pin1 inhibitors for treatment of diseases such as cancer, there are important considerations to be taken note of. Much interest has been put forth on identifying Pin1 inhibitors that target the PPIase domain as well as its active site. As suggested in this review, the binding affinity of Pin1 inhibitors could be affected by the substrate that is bound to the WW domain. Therefore, binding affinity studies for Pin1 inhibitors screening should consider the substrate effect upon its binding to the WW domain, and how this effect could alter the Pin1 inhibitors binding to the PPIase domain or its active site. Of interest, a very recent study by Momin et al. (116) using MD analysis suggests that substrate sequence can influence the eventual outcome of Pin1 function by differential triggering of Pin1 allosteric changes after substrate binding. This is explained by the substrate sequence-dependent allostery that affects the type of residue to residue contact within the interdomain interface. This led to the authors to suggest that drugs targeting the specific geometry of the interdomain interface at a specific Pin1 substrate binding could lead to better treatments of specific diseases. Whether these MD simulations could be translated to *in vivo* circumstances are yet to be determined and could be explored in future drug development for Pin1 inhibitor.

This potential substrate-dependent Pin1 activity could be important in future studies. This is highlighted in the lack of depth in the field of Pin1 research thus far. Most studies done on Pin1 mechanism focuses on just a few biological substrates of Pin1. As suggested in this review, the vast repertoire of Pin1 functional substrates and the different biological processes it could affect do point to the fact that Pin1 might behave differently in the presence of different substrate. This difference in behavior could affect its structural dynamics that makes identifying a functional Pin1 inhibitor difficult for different types of diseases. Therefore, to advance the research of the role of Pin1 in the pathogenesis of various diseases, more emphasis on the diversity of Pin1 substrates being tested must be done. As mentioned previously, Pin1 inhibitor screening could be done in the presence of its effector substrate to evaluate the efficacy of the Pin1 inhibitor in affecting the phenotype of the disease being studied. In addition, studies looking into how Pin1 cooperation with kinase in exerting its isomeric activity could be conducted to investigate if the proposed multimeric model holds true, and how the subsequent findings could affect the considerations when developing Pin1 inhibitors. Indeed, studies thus far do suggest that Pin1 is not a lone ranger, and it always needs a helping hand to exert its function. To advance the development of drugs to highlight the impact of Pin1 as a cancer therapy target, both domains should be considered during the drug development process for the field to realize the potential of Pin1 as a cancer therapeutic target.

## Author contributions

All authors listed have made a substantial, direct and intellectual contribution to the work, and approved it for publication.

### Conflict of interest statement

The authors declare that the research was conducted in the absence of any commercial or financial relationships that could be construed as a potential conflict of interest.
